# Exploring the Interaction of Tumor-Derived Exosomes and Mesenchymal Stem Cells in Tumor Biology

**DOI:** 10.3390/ijms26073095

**Published:** 2025-03-27

**Authors:** Konstantinos S. Papadopoulos, Penelope Korkolopoulou, Christina Piperi

**Affiliations:** 1 Department of Plastic and Reconstructive Surgery, 401 General Military Hospital of Athens, 11525 Athens, Greece; 2 First Department of Pathology, Medical School, National and Kapodistrian University of Athens, 11527 Athens, Greece; pkorkol@med.uoa.gr; 3 Department of Biological Chemistry, Medical School, National and Kapodistrian University of Athens, 11527 Athens, Greece

**Keywords:** mesenchymal stem cells, cancer, tumor-derived exosomes, angiogenesis, TME, immune response, adipose-derived stem cells

## Abstract

Exosomes are actively produced extracellular vesicles, released from different cell types, that exert important regulatory roles in vital cellular functions. Tumor-derived exosomes (TDEs) have received increasing attention because they enable intercellular communication between the neoplastic and non-neoplastic cells present in the microenvironment of tumors, affecting important functions of different types of mesenchymal stem cells (MSCs) with the ability to self-renew and differentiate. MSC-derived exosomes (MSC-exos) carry a variety of bioactive molecules that can interact with specific cellular targets and signaling pathways, influencing critical processes in tumor biology, and exhibiting properties that either promote or inhibit tumor progression. They can regulate the tumor microenvironment by modulating immune responses, enhancing or suppressing angiogenesis, and facilitating tumor cells’ communication with distant sites, thus altering the behavior of non-cancerous cells present in the microenvironment. Herein, we explore the main functions of TDEs and their intricate interactions with MSC-exos, in terms of enhancing cancer progression, as well as their promising clinical applications as tumor microenvironment modulators.

## 1. Introduction

Extracellular vesicles (EVs) present a constantly expanding class of micro- and nanoparticles, produced by cells as key mediators of cellular communication, containing nucleic acids, proteins, or lipids. While their existence has been known about for years, they were often considered byproducts of apoptosis or cell death [1].

All EVs share a common ability to act outside the cell, either towards other distant target cells or by diffusion of their cargoes to adjacent cells in a paracrine manner. At the same time, the absence of intercellular contact is typically required. The discovery of a gradually increasing number of EVs in recent decades has resulted in the need to code and classify them, according to their function, properties, and differential characteristics, such as size, site of biosynthesis, time of exocytosis, and type of cargo. Although this is a difficult and constantly evolving process, two main types can be distinguished, namely, the ectosomes formed on the cell membrane, which are often referred in the literature as microvesicles, nanoparticles, oncosomes, etc., and the exosomes.

Exosomes present a broad category that describes intraluminal vesicles (ILVs) arising from the engulfment of late endosomes to create multivesicular bodies (MVBs) through an intracellular sequence of mechanisms mobilized by a multi-unit system called endosomal sorting complex required for transport (ESCRT). The subunit ESCRT-0 recruits ubiquitinated proteins and lipids to be used by subunits ESCRT-1 and ESCRT-2 in order to synthesize ILVs. Finally, after relaxation of the cytoskeleton, the vesicles reach the ESCRT-3 subunit to fuse with the membrane and be subsequently secreted. ILVs, when released extracellularly, are characterized as exosomes and will act on nearby or distant target cells [2].

The regulation of exosome production and secretion is still largely unclear, but Rab family genes (*Rab*, *Rab11*, *Rab27A*, *Rab27B*, and *Rab35*) [3] have been found to actively participate at many different time-points of their secretion in different cell lines [4].

Upon their release, EVs interact with only specific target cells and not with any cell type. For example, platelet-secreted EVs have been found to react with endothelial cells and macrophages but not with neutrophils [5]. During exocytosis, some EVs may rupture and allow their components to be released extracellularly. Several growth factors such as TGF-β and interleukins, e.g., interleukin-1β (IL-1β), can further bind to receptors present on neighboring cells to elicit a rapid response [6]. EVs that retain their structure can travel through body fluids, including blood, lymph, and cerebrospinal fluid, eliciting responses in distant organs and target cells [7].

Exosomes’ metabolism depends on blood concentration–time profiles, with pharmacokinetic analysis showing that circulating exosomes are rapidly metabolized with a half-life of approximately 2–30 min. They can be further phagocytosed by macrophages in the spleen, liver, and lung, regardless of their cells of origin or isolation methods [8]. Exosomes contain a variety of cargoes including DNA, lipids, proteins, mRNA, microRNA, and lncRNA (long non-coding RNA), with the latter being of particular interest. The ExoCarta database includes most of the possible cargoes that can be contained in exosomes, which, however, depend mainly on the type of producing cell, its interaction with neighboring cells, or the extracellular environment [9].

Based on the current scientific literature, exosomes are characterized as EVs of smaller diameter, 50–100 nm, with limited ability to adhere to the cell membrane. They undergo delayed exocytosis because they are bound within multivesicular bodies (MVBs) and may be characterized by the expression of non-specific markers CD63, CD9, and CD81 [10]. The increasing number of studies unraveling new vesicles and their functions has created the need to establish guidelines on nomenclature, techniques used for characterization, and the correct isolation methods of EVs to maintain both the reproducibility and the quality of respective experiments. These guidelines are referred to as the Minimal Information for Studies of Extracellular Vesicles (MISEV2018) and include all the necessary directions that need to be followed in order to detect the exact nature of the particles for valid results [11].

Commonly, exosomes released from neoplastic cells are larger than those from normal cells due to cancer cells heterogeneity [12]. Emerging research data indicate that tumor-derived exosomes (TDEs) are often involved in the intercellular communication of neoplastic and non-neoplastic cells present in the tumor microenvironment. Many different types of malignant cells synthesize and secrete exosomes with specific cargoes, including proteins (and oncoproteins), lipids, RNA, and DNA, that can provide important insights into the molecular signature of each tumor. In the TME, different sub-clones of cancer cells are present due to overexpression of various genes associated with tumor growth and local invasion. Still, they can also excrete the modified vesicles associated with chromosomal deletion of specific regions in metastatic disease. For this reason, cancer cell-derived exosomes can be either small (100–400 nm) or large (1–10 μm) oncosomes, depending on their size and cargo of several macromolecules (Table 1) [13].

Recent evidence indicates that TDEs have a pivotal role in tumor biology, influencing various aspects of cancer progression through various mechanisms including intercellular communication, angiogenesis, metastasis promotion, immune modulation, therapeutic resistance, and tumor microenvironment modifications [14]. In this review, we explore the diverse functional roles of TDEs, their interaction with mesenchymal stem cells (MSCs), and their potential applications in improving cancer diagnosis and treatment strategies.

## 2. Functions of Tumor-Derived Exosomes (TDEs)

During the last few years, there has been an increasing effort to elucidate the impact of exosomes derived from cancer cells on the growth and metastasis of mesenchymal or epithelial tumors. TDEs are extracellular vesicles, larger than typical exosomes, that are formed and secreted into the extracellular space, and which contain a complex cargo of bioactive molecules, including oncoproteins, lipids, RNA, and DNA. However, it is important to highlight that the molecular cargoes of cancer-derived EVs comprise the oncogenic potential of cancer cells and the term “oncosomes” does not simply refer to their cellular origin [15]. In addition, the cargoes and functions of large and nanosized EVs with the same origin may also differ [16]. Nevertheless, even though they differentiate from typical exosomes, there is no consensus on the precise terminology of these vesicles [17].

### 2.1. Effects on Intercellular Communication

**Figure 1:**
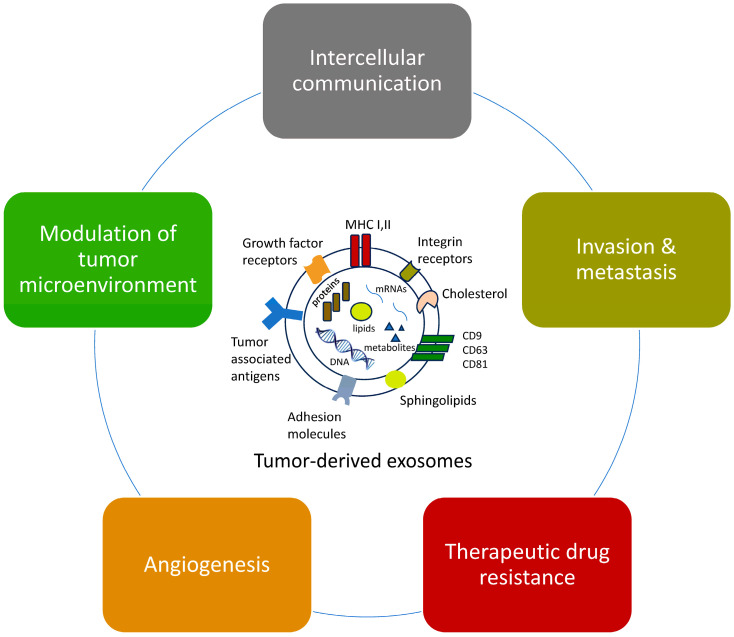
Tumor-derived exosomes are involved in the modulation of tumor microenvironment, intercellular communication, angiogenesis, invasion, and metastasis, as well as drug resistance.

TDEs facilitate tumor cell communication with the microenvironment by transferring oncogenic molecules, such as nucleic acids and proteins, to adjacent cells, altering their behavior and promoting tumor progression (Figure 1). Rhabdomyosarcoma exosomes, for example, enhance the differentiation and proliferation of both neighboring fibroblasts and cells of the sarcoma itself, promoting angiogenesis, fibroblast migration, and infiltration, ultimately resulting in tumor growth [18]. In addition, the characteristic *PAX3-FOXO1* gene fusion of alveolar rhabdomyosarcoma (aRMS) appears to be associated with miR-486-5p, which induces changes in the exosomal contents originating from myoblasts and acts as a pro-oncogenic signal to promote migration, infiltration, and colony formation [19].

Liposarcoma exosomes (LPS) have been shown to stimulate tumor-associated macrophages (TAMs) to release the pro-inflammatory cytokine IL-6, inducing tumor proliferation and metastasis with miR-92a-3p and miR-25-3p [20].

In myxofibrosarcoma (MFS), a malignant soft tissue tumor, EVs with miR-1260b affect normal surrounding fibroblasts and enhance tumor growth through downregulation of the tumor suppressor gene protocadherin 9 (*PCDH9*). In addition, overexpression of miR-1260b is associated with a more aggressive radiographic image, characteristic of invasive MFS [21].

### 2.2. Promotion of Metastasis

Exosomes can also modulate the metastatic spread of cancer by preparing the pre-metastatic niche since they can travel to distant organs, modifying the local environment to create a favorable niche for incoming tumor cells. They can also enhance tumor cell motility by transferring molecules that increase the invasive properties of cancer cells. This migratory potential is orchestrated by mutations on the TGF-β pathway that promote epithelial-to-mesenchymal transition (EMT) and metastasis through the suppression of E-cadherins and the induction of metalloproteinases (MMPs). This will eventually distort the extracellular matrix (ECM) to create a congenial environment for the tumor [22].

Tumors such as osteosarcoma (OS) affect mesenchymal cells in various ways, by increasing oxidative stress and lactate levels due to exposure to the tumor microenvironment, which can enhance the metastatic potential of osteosarcoma [23]. EVs from OS cell lines were shown to affect normal cells in the TME by enhancing their proliferation and migration capacity as well as by increasing their survivability and adhesion. These EVs exhibit an oncogenic potential, regardless of the presence of cancer cells and associated factors, including IL-6, TNF-α, and TGF-β with de novo MMP-9 and TNF-α expression [24]. Another study with similar results demonstrated that miR-21-5p and miR-148a, found in OS exosomes, could induce immortalization of the umbilical vein endothelial cells (HUVECs) [25]. As anticipated, these tumor exosomes consistently promoted tumor growth and metastasis through immunosuppression and interaction with the TME. The specific effects of exosomes on OS were consistent among many different studies and were in favor of tumor growth [26].

Cancer cell exosomes may influence the activity of mesenchymal cells through reprogramming and the acquisition of new properties. Adipose-derived stem cells (ADSCs) are pluripotent cells with innate angiogenic and proliferating abilities that stem from their exosomes. These cells are able to alter their functions depending on the molecular signals they are exposed to [27]. ADSCs from patients with prostate cancer can undergo mesenchymal–epithelial transition (MET), express specific markers, and mimic the primary tumor when exposed to tumor cell exosomes in vivo that contain oncomiRs such as miR-130b, miR-125b, and miR-155 [28]. Other cancer cell types (e.g., breast and ovarian cancer cells) secrete exosomes that can make ADSCs to resemble myofibroblasts (tumor-associated myofibroblast), possibly through a paracrine mechanism on the neighboring stromal cells of their microenvironment, thus influencing local tumor progression. The use of tumor exosomes was further shown to be associated with the increased expression of growth factors including TGF-β [29,30].

### 2.3. Effects on Axonogenesis

A particular function of TEDs in head and neck tumors is their involvement in tumor innervation. Exosomes were demonstrated to induce axonogenesis in a rat pheochromocytoma (PC12) cell line, a process of neo-innervation that was characterized by the formation of disordered nerve fibers, described as nerve twigs that were positive for β-III tubulin. This process was shown to be mediated by exosomes of cancer cells and not by exosomes of normal cells, differing from the perineural invasion seen during tumor extension [31].

### 2.4. Effects on Angiogenesis

Tumors have the potential to serve as “angiogenic switches”, altering their anti-angiogenic environment to a pro-angiogenic one. This function is facilitated by exosomes that promote new blood vessels’ formation by delivering pro-angiogenic factors such as VEGF and miRNAs to endothelial cells [32].

Tumor cells may also release proteins and TDEs containing tetraspanin 8, which can trigger transcriptional changes in endothelial cells, leading to the upregulation of pro-angiogenic genes and enhanced endothelial cell proliferation [33]. Other exosomal cargoes such as the oncogenic EGFR may be secreted from neoplastic cells to affect normal endothelial cells [34].

A study of A431 carcinoma cells subjected to hypoxia or reoxygenation showed that they displayed increased angiogenic and metastatic capabilities and stronger intercellular and cell-to-extracellular matrix adhesion, as well as enhanced invasiveness. Although the majority of these secreted proteins were of cytoplasmic or membrane origin [35], the hypoxic environment acts as the trigger for malignant cells to produce hypoxia-induced exosomes with miR-210, a process possibly mediated by HIF-1a [36].

### 2.5. Effects on Therapeutic Drug Resistance

Exosomes can contribute to therapeutic resistance by carrying as well as removing chemotherapeutic drugs from cancer cells, thus reducing drug efficacy. They can also transfer drug resistance-related proteins and miRNAs to sensitive cells, conferring resistance to treatment. In addition, some tumors are composed of cancer stem cells (CSCs) that can also produce exosomes and other EVs [37], contributing to tumor heterogeneity [38].

CSCs expressing CD133 in sarcomas or other cancer types also exhibit high levels of tumor-related genes and may be directly related to chemotherapy resistance and increased metastatic potential through various pathways [39]. For example, the chemotherapeutic resistance of CSCs has been studied in MCF-7 breast cancer cells. These CSCs had a noticeable high CD44 and low CD24 expression, along with Oct4 expression. Additionally, they could achieve anchorage-free growth in the form of tumor spheres that the chemotherapeutic drug paclitaxel was unable to inhibit [40].

Furthermore, exosomes can be armed with specific molecules bearing anti-tumor properties and can target cancer cells and CSCs. The use of miR-503-3p in ADSC-exos was shown to potentially suppress the initiation and progression of CSC growth, inhibit the formation of the characteristic spheroid formations by up to 69%, and block tumor growth in vivo. These molecules seem to exert some degree of negative regulatory action against characteristic genes of the CSCs such as *Nanog*, but without consistently suppressing them [41]. Of particular interest, CSCs can be reprogrammed into non-tumorigenic cells by exosomes released from osteogenically differentiated ADSCs (OD-EXOs) containing specific cargoes capable of differentiating CSCs to be less resistant to systemic therapies [42].

### 2.6. Modulation of the Tumor Microenvironment

The tumor microenvironment (TME) consists of a variety of different cell types, including stromal, vascular, and immune cells, as well as signaling molecules within the extracellular matrix, serving similar biological roles among cancer types ranging from angiogenesis, metastasis, and chemoresistance to immune regulation.

Low pH conditions in the TME characterize malignant tumors and may affect the release as well as the uptake of exosomes. In a study of melanoma cancer cells, the uptake of exosomes by cells of metastatic origin was higher compared to those released from primary tumors or normal cells. Moreover, their uptake by melanoma cells was inhibited after exposure to proton pump inhibitors and an increase in pH [43].

Exosomes influence various cells of the tumor microenvironment, including endothelial cells, fibroblasts, and immune cells, promoting a pro-tumorigenic environment. They can induce fibroblast activation, extracellular matrix remodeling, and suppression of anti-tumor immune responses.

Exosomal integrins participate in the organotropism of metastasis and may be used to detect organ-specific metastasis. Proteomic analysis of TDEs revealed distinct patterns of integrin expression, with α6β1 and α6β4 shown to be involved in lung metastasis, while exosomal integrin αvβ5 was correlated with liver metastasis. Exosomes derived from liver, lung, and brain tumor cells were studied in mice as well as humans and detected to fuse with normal cells at their intended destination. Their uptake was shown to activate Src phosphorylation and the expression of pro-inflammatory gene S100, thus playing an important role in the formation of the pre-metastatic niche [44].

Tumor-associated macrophages are mainly found within the tumor mass and interact with the TME by promoting or inhibiting tumor growth. The balance between the pro-tumorigenic and anti-tumorigenic functions is complex and relies on the cell phenotype at any given time. TAMs, like normal macrophages, can present with an M1 or M2 phenotype, affecting the tumor and its environment in various ways through exosomes. In the TME, M1-polarized TAMs may exhibit immunoinductive and tumor-suppressive behavior via specific EVs. These vesicles increased the pro-inflammatory cytokines such as IL-1β, TNF-α, and IL-6, and stimulated the apoptosis of osteosarcoma (OS) and melanoma cancer cells by increasing the activation of caspase-3 and caspase-7 [45]. On the contrary, immunosuppressive M2 polarization occurs at later stages and induces tumor growth. M2-TAM exosomes, rich in miR-221-3p, downregulate the SOCS3/JAK2/STAT3 axis, which promotes the malignant behavior of OS cells [46]. In another study, TAM-exosomes caused an increase in lncRNA LIFR-AS1, which suppresses miR-29a, a contributor to apoptosis and, thus, affects the growth and metastatic ability of OS through the miR-29a/NFIA pathway [47].

Exosomes can also drive the interaction of fibroblasts and cancer cells to advance sarcoma growth. The so-called CAFs (cancer-associated fibroblasts) are involved in the remodeling of the ECM and the generation of the pre-metastatic niche (Table 2). The exosomes of CAFs seem to depend on the type of TME of each tumor. For example, in the osteosarcoma TME, exosomal SNHG17 is involved in intercellular communication since it affects miR-2861, regulates the expression of MMP-2, and provides suitable conditions for the expansion and metastasis of neoplastic cells [48]. Additionally, EVs from OS cells, rich in TGF-β1, mediate fibroblast differentiation in the area of lung metastasis and prepare the ground for the metastatic niche [49].

## 3. TDEs Interact with Mesenchymal Cell Exosomes (MSC-exos) Within the Tumor Microenvironment

Research data suggest that exosomes from mesenchymal cells (MSC-exos) affect tumor growth through the induction of different complex signaling pathways and may promote tumorigenesis or act as tumor suppressors. Tumor growth, which relies on growth factors such as PDGFR, EGFR, VEGF, etc., is promoted by the activation of the respective receptors through various pathways such as Akt, PKC/PKB, and MAP kinases [50]. Although the number of studies with ADSCs in relation to malignant tumors is quite limited, it has been shown that the addition of ADSC-exos in breast cancer cells promotes tumor growth and migration through the Wnt/b-catenin signaling pathway along with *Dkk1* and *Axin2* target genes [51].

Another study about human ADSCs and dermatofibrosarcoma cells detected a significant tumor progression through the induction of growth factors and angiogenesis that was suggested to be mediated by exosomes [52]. Similar results were obtained upon investigation of ADSC-exos impact in osteosarcoma both in vitro and in vivo. ADSC-exos were shown to promote metastasis through the amplification of the *COLGALT2* gene, which is also overexpressed in the osteosarcoma cells themselves. In addition, there was an overexpression of vimentin and MMP2/9 metalloproteinases and induction of the EMT, inducing more aggressive tumor behavior [53].

Accordingly, in ovarian tumors, ADSC-exos derived from omental adipocytes promoted progression and peritoneal metastasis via the MAPK, cyclin F, and other signaling pathways [54].

On the contrary, treatment with ADSC-exos in hepatocellular carcinomas was found to increase the tumor suppressor effect of NK T-cells, reduce the bulk of the tumor, and induce changes in the histopathology that correlated with lower tumor grade [55].

Studies focused on BMSC-exos in various cancer types demonstrated variable results, for example, overexpression of the exosomal protein lymphocyte cytosolic protein 1 (LCP1) from BMSCs promoted osteosarcoma growth via the JAK2/STAT3 pathway, while simultaneously suppressing the tumor suppressor miR-135a-5p [56]. The combination of ΒMSC-exos and SGC-7901 cells (subcutaneous tumor model of gastric cancer cell line) increased the ability of cancer cells to be transplanted, along with promoting a stronger proliferation potential through ERK 1/2 pathway activation, whereas the combination with cells of multiple myeloma led to enhanced survival via the p38, the c-Jun N-terminal kinase p53, and Akt pathway [57,58]. On the contrary, when BMSC-exos were injected into hepatocellular tumors, Kaposi sarcomas, and ovarian tumors in vitro and in vivo, resulted in increased cell death and reduction in the tumor, respectively [59]. A similar tumor suppressive effect of MSC-exos was detected in breast cancer cells, where the miR-16 cargoes mediated inhibition of VEGF and elicited an anti-angiogenic effect [60].

Altogether, research data indicate that exosomes and overall EVs produced by mesenchymal cells may have controversial to unpredictable effects that are associated with the origin and identity of their cargo when interacting with a tumor and its respective microenvironment. MSCs have the ability to absorb TDEs in a similar manner to the one in which they absorb other exosomes and can be altered to stimulate tumor growth via increased macrophage infiltration [61]. This process dynamically changes the chemokine profile of normal MSCs and turns them to tumorigenic cells that produce large amounts of CCL2 and CCL7 that act as ligands for macrophage recruitment.

As stated before, CAFs also play a vital role in tumor progression but their regulation and origin are still uncertain. Recent data suggest that TDEs may engage in CAF formulation and differentiation through the mechanism of endothelial-to-mesenchymal transition. Cancer cells that are depleted of exosomes fail to exploit their effects on fibroblasts and are incapable of promoting growth of the tumor-associated stroma [62]. In another study, exosomal TGF-β and other cargoes from TDEs were shown to promote the formation of CAFs from endothelial cells with normal MSCs, exerting negative feedback on the trans-differentiation of CAFs and, subsequently, alleviating tumor growth and metastasis [63].

## 4. Clinical Applications of Tumor-Derived Exosomes

With biotechnology currently thriving, researchers are trying to use new molecules as diagnostic markers as well as carriers for specific biomolecules such as micro-RNAs that will, ideally, have predictable and beneficial anti-tumor effects and enable the therapeutic use of exosomes. TDEs, being essential for communication between cells, are implicated in cell proliferation, signaling and differentiation, regulating oncogenesis, migration, and metastasis.

A variety of methods have been used for the isolation and characterization of exosomes, with the gold standard technique involving sequential centrifugation and ultracentrifugation to pellet exosomes. In addition, size-exclusion chromatography (SEC) can be applied to separate exosomes based on their size without, however, affecting their integrity, while precipitation-based kits using polymers can be used to precipitate exosomes from biofluids. Several microfluidic devices also employ lab-on-a-chip platforms with immunoaffinity or size-based separation for high-throughput capture of exosomes. Moreover, antibodies targeting exosome-specific markers (e.g., EpCAM, CD9, CD63, and CD81) to selectively bind TDEs can be used, as well as synthetic oligonucleotides (aptamers) that specifically recognize tumor exosomal markers [18,20,21,22,24,25]. Upon isolation, several techniques can be employed to analyze exosomes and detect cancer-specific molecular signatures. Nanoparticle tracking analysis can measure the size and concentration of exosomes while their structural details can be elucidated by scanning and transmission electron microscopy (SEM/TEM). Exosomal protein markers specific to tumors can be detected by Western blotting, mass spectrometry, and ELISA, while exosomal RNA can be detected by qPCR and RNA sequencing [64].

Importantly, TDEs can be detected in liquid biopsies, providing significant clinical insights for the early diagnosis of tumors and minimizing the invasive routine tissue biopsies along with their complications. The concentration of TDEs in biological fluids varies significantly depending on the type and stage of tumor as well as on the patient’s condition. It has been estimated that the total exosome concentration in human plasma or serum ranges from 10^8^ to 10^12^ particles per ml while TDEs may constitute 10–50% of this total exosome amount, being 10–100 times higher in aggressive cancers. Increased TDE levels have been detected in lung, breast, and pancreatic cancers, as well as in glioblastoma. Their abundance is further increased in the advanced tumor stage, hypoxia, immune stress, and therapeutic schemes [64].

Exosomal and EV miRNAs that were directly associated with specific tumors have been further assigned to their molecular signature with diagnostic significance. Another study investigated similar vesicles identified by flow cytometry in the circulation and described them as large oncosomes (1–10 μm) that originate from the bulk of prostate tumor protrusions that can interact with endothelial cells, fibroblasts, and the other tumor cells. MMP-9, MMP-2, and other molecules such as caveolin-1 and ADP-ribosylation factor 6 were identified as cargoes of those large oncosomes [65]. Minciacchi et al. reported that there was a 25% difference in the cargoes of large and nano-sized oncosomes after quantitative proteomics, with molecules involved in invasion and tumor progression, like cathepsin proteases, tetraspanins, and growth factors, being abundant in nano-sized EVs. Further studies are needed to elucidate the specificity of these cathepsin-containing vesicles and to ascertain whether they could be considered as potential diagnostic markers [16].

Recent data have shown that not only miRNAs but many different molecules such as mRNA and lncRNA can be detected by next generation sequencing techniques (NGS) through analysis of the entire transcriptome (whole transcriptome RNA-sequencing) as well as by using novel techniques such as employing 3D-printed integrated microfluidic chips [66]. Finally, exosomes themselves can be enriched through surface markers or nano-flow cytometry for more reliable results and enhance the sensitivity of liquid biopsies in finding specific mutations and serve as circulating RNA biomarkers [67].

In an extensive analysis of mesothelioma cell exosomes, several tumor-specific protein cargoes were found that could serve as diagnostic targets. Chondrosarcoma exosomes carrying specific lncRNAs modulate the RAMP2-AS1/miR-2355-5p/VEGFR2 axis promoting angiogenesis, with RAMP2-AS1 being a potential biomarker of interest [68,69].

High expression of extracellular miR-675 has been detected in metastatic osteosarcoma cells but not in their non-metastatic counterparts [70]. In addition, extracellular miR-143, mainly found within exosomes, is involved in the biology of osteosarcoma metastasis through the regulation of the metalloproteinase MMP-13, indicating a potential prognostic marker [71].

Another interesting application of exosomes is their use as transporters of therapeutic molecules for targeted treatment in oncology. Investigation of the molecular composition of various exosomes revealed similar rapid clearance and distribution after intravenous administration, regardless of their unique protein or lipid cargo, potentially limiting their use as systemic anticancer therapies. On the contrary, when delivered intralesionally, the exosomes remained bound to the tumor tissue to a significantly greater extent. Furthermore, experiments in immunocompromised mice revealed the importance of non-specific immunity together with the complement protein C5 in the exosome clearance rate [72].

Exosomes that have been used as carriers of functional miRNAs, such as synthetic miR-143, can block the invasive and metastatic properties of osteosarcoma cells. Of interest, exosomal miR-143 increased after the administration of the synthetic analog into cells, while exosomes transported miR-143 to the respective subcellular location to reach its mRNA target in recipient cells more efficiently than other carriers such as liposomes. This evidence indicates that exosomes transported between cells change the expression of genes and may affect specific functions of osteosarcoma cells through the delivery of miRNAs [73].

Exosomal miR-15a directly targets GATA2 and the GATA2/MDM2 axis, which is overexpressed in OS, and drives continuous cell proliferation. Inhibition of this overexpression interrupts the dysregulation of the cell cycle and slows the onset of OS [74]. The selectivity of miR-15a for this pathway may have potential clinical applications since this overexpression is often an indicator of resistance to adjuvant therapies. Biotechnologically engineered exosomes rich in miR-15a can be potentially used in sarcomas that characteristically overexpress MDM2, such as well-differentiated and dedifferentiated liposarcomas [75,76].

Additional data showed that ADSC-derived exosomes are also an efficient in vivo delivery vehicle and exosomal miR-138-5p is a potential therapeutic molecule for the treatment of bladder cancer. ADSC-exos could infiltrate tumor tissues and release miR-138-5p to inhibit tumor growth [77]. Furthermore, engineered ADSC-EVs can secrete and transport miR-101, a microRNA present at very low levels in metastatic OS cells, limiting lung metastases [78]. Exosomal miR-1913 was shown to restrict OS growth through downregulation of NRSN2 [79], while miR-150-loaded exosomes target IGF2BP1 with similar effects in OS cells [80].

In a study of 11 cancer cell lines, TRAIL (TNF-related apoptosis-inducing ligand) was used in MSC-EVs to induce apoptosis in a dose-dependent manner without significant cytotoxicity [81]. Similar results were observed in the study by Lou et al. where ADSC-exos with miR-122 were used to increase the chemosensitivity of hepatocellular tumors to sorafenib in vivo [82]. Another investigation showed that the tumor suppressor miR-145 in both ADSCs and exosomes exerted a suppressive effect on prostate cancer cells by promoting apoptosis through inhibition of the anti-apoptotic protein Bcl-xL [83].

Tumor-derived exosomes can, therefore, enable personalized medicine and customized treatment (Table 3). The molecular content of TDEs can give important information on the genetic and molecular profile of a patient’s tumor, enabling personalized treatment strategies, customized to the specific characteristics of the cancer. Moreover, they can be used in exosome-based vaccines since they contain tumor antigens for stimulation of the immune response to cancer cells. This approach aims to train the immune system to recognize and attack cancer cells more effectively.

Unraveling the content of exosomes can also help identify mechanisms of resistance to therapy and enable the design of strategies to prevent or overcome resistance. Targeting exosome-mediated resistance pathways in combination with conventional therapies may improve treatment efficacy. The use of exosomes in liquid biopsies further allows for continuous monitoring of cancer patients with minimal invasiveness. This approach can detect changes in tumor dynamics and genetic mutations, providing insights into the evolving nature of the disease.

## 5. Conclusions

Extracellular vesicles are undoubtedly one of the main points of interest in current studies focused on elucidation of the molecular nature of cancer. Their applications range from non-invasive diagnostics and real-time monitoring of treatment responses to targeted drug delivery and personalized medicine. The interactions between malignant and mesenchymal stem cells seem to play a pivotal role in tumor biology and progression, and need thorough investigation. Nevertheless, this relationship may be the key to unlocking the unique properties of exosomes that will help clinicians to improve the accuracy of cancer diagnosis, provide tailored treatments to individual patients, and develop innovative therapeutic strategies. Tumor-derived exosomes and studies on oncosomes hold promise in revolutionizing the understanding of cancer evolution and clinical management. It is crucial, however, to properly evaluate the plethora of information collected by numerous studies in order to specifically determine their clinical significance in the future.

## Figures and Tables

**Table 1:** Similarities and differences between different types of EVs.

Characteristics	Ectosomes	Exosomes	Large Oncosomes
Diameter range	100–350 nm	50–100 nm	1–10 μm
Accumulation site	Plasma membrane	Intracellular MVBs	Plasma membrane
ESCRT complex utilization	Yes (Partially)	Yes	Yes (Partially)
Release procedure	Pinching off	Exocytosis	Pinching off
Release timing and amount	Early—high amount	Late—controlled	Early—high mount
Possible markers	TyA, C1q	CD63, CD61, CD81, CD9	CK18, GAPDH, HSPA5

**Table 2:** Functions of tumor-derived exosomes (TDEs).

Type of Cancer Cells	Exosomal miRNA	Target Cells	Mechanism of Action	Effects	References
RMS	miR-486-5p	CAFs, fibroblasts, myoblasts	Angiogenesis, migration, differentiation and proliferation of fibroblasts and cancer cells	Tumor invasion and increased metastatic capacity	[18,19]
LPS	miR-486-5p miR-92a-3p	TAMs	Increased IL-6	Tumor proliferation and increased metastatic capacity	[20]
MFS	miR-1260b	Fibroblasts	Down-regulation of PCDH9	Tumor proliferation	[21]
OS	EVs	MSCs	Effect on the tumor microenvironment TNF-α, IL-6, TGF-β, MMP-9	Oncogenic potential in the absence of cancer cells	[23,24]
OS	miR-148a miR-21-5p	HUVECs	Effect on the tumor microenvironment	Induction of immortality in target cells	[25]
Prostate	miR-125b, miR-130b miR-155	ADSCs	Expression of epithelial, neoplastic and angiogenic tumor markers	Mesenchymal-epithelial transition	[28]
Breast	-	ADSCs	Overexpression SDF-1, VEGF, CCL5, TGF-β, SMAD2	Convert ADSCs to myofibroblasts	[29]
Ovaries	-	ADSCs	Overexpression SDF-1, TGF-β, SMAD2	Convert ADSCs to myofibroblasts	[30]

RMS: rhabdomyosarcoma, LPS: liposarcoma, MFS: myxofibrosarcoma, OS: osteosarcoma, CAFs: cancer-associated fibroblasts, HUVECs: human umbilical vein endothelial cells, ADSCs: Adipose-derived stem cells and TAMs: tumor-associated macrophages.

**Table 3:** Mesenchymal cell exosomes affect the TME and tumor progression.

Origin of Exosomes	Target Cells	Mechanism of Action	Effects	Reference
ADSC-exos	CSCs	Suppression of spheroid formation of CSCs by miR-503-3p	Tumor suppression	[40]
ADSC-exos	CSCs	Increased expression of APLP, RUNX2, and BGLAP	Reprogramming of CSCs into non-tumorigenic cells	[41]
ADSC-exos	Breast cancer	Induction of the Wnt/b-catenin signaling pathway	Tumor progression	[51]
ADSC-exos	DFSP	Overexpression of VEGF, HGF, and bFGF, PDGFRB, COL1A1	Tumor progression	[52]
ADSC-exos	OS	Vimentin and MMP 2/9 overexpression, EMT induction	Tumor progression	[53]
ADSC-exos	Ovarian cancer	FOXM1, Cyclin F, KIF20A, and MAPK	Tumor progression	[54]
ADSC-exos	Hepatocellular carcinoma	Enhancement of NK T-cell activity	Tumor suppression	[55]
BMSC-exos	OS	LCP1, JAK2/STAT3 pathway, miR-135a-5p suppression	Tumor progression	[56]
BMSC-exos	Stomach cancer	Activation of the ERK 1/2 pathway	Tumor progression	[57]
BMSC-exos	Multiple myeloma	p53, p38 and Akt pathways	Tumor progression	[58]
BMSC-exos	Hepatocellular carcinoma, ovarian cancer, Kaposi’s sarcoma	DIRAS3, RBL2, RBL1, CDKN2B, CDKN1A, CCNE1, SKP2, CCND2, CUL3, GAPDH	Tumor suppression	[59]
MSC-exos	Breast cancer	miR-16 cargoes of exosomes inhibit VEGF	Tumor suppression	[60]
ADSC-exos	Bladder cancer	Tissue invasion and tumor growth suppression via miR-138-5p	Tumor suppression	[77]
ADSC-EVs	OS	They limit the development of pulmonary metastases through miR-101	Tumor suppression	[78]
ADSC-exos	OS	Negative regulation in NRSN2 by miR-1913	Tumor suppression	[79]
ADSC-exos	OS	Negative regulation of IGF2BP1 by miR-150	Tumor suppression	[80]
ADSC-exos	Hepatocellular cancer	Chemosensitivity of hepatocellular tumors to sorafenib via miR-122	Tumor suppression	[81]
ADSC-exos	Prostate cancer	Enhancement of apoptosis through Bcl-xL inhibition via miR-145	Tumor suppression	[83]

CSCs: cancer stem cells, DFSP: dermatofibrosarcoma, ADSC: adipose tissue stem cells, OS: osteosarcoma, and BMSC: bone marrow stem cells.
